# Ethical prompting: toward strategies for rapid and inclusive assistance in dual-use AI systems

**DOI:** 10.3389/frai.2025.1646444

**Published:** 2025-09-15

**Authors:** Joan Farnós, Alger Sans Pinillos, Vicent Costa

**Affiliations:** ^1^Barcelona Supercomputing Center (BSC), Barcelona, Spain; ^2^Artificial Intelligence Research Institute (IIIA-CSIC), Cerdanyola del Vallès, Spain

**Keywords:** aid systems, bias, artificial intelligence in defense, autonomous command and control systems, blind obedience to orders, disability, dual-use dilemma, human-centered AI

## Abstract

Monitoring technologies initially developed for individuals with disabilities carry inherent dual-use risks, especially evident in conflict or emergency scenarios. This article examines the dual-use dilemma posed by technologies whose civilian design objectives can unintentionally facilitate harmful applications in defense contexts. Specifically, we analyze the ethical risks associated with using civilian-generated data and systems, originally intended to enhance care and assistance, for military purposes without adequate safeguards. We argue that effective and ethically sound technological infrastructures require optimized and ethically-informed prompting strategies. These strategies must clearly define how data and system prompts are structured, reducing deployment biases, particularly against vulnerable populations.

## 1 Introduction

While defense-oriented research often raises legitimate ethical concerns, it also has considerable potential to benefit the civilian sector, particularly in emergency or extreme vulnerability situations. In this regulatory sense, dual-use technologies are defined as ‘software and technology that have the potential to be used for both civil and military purposes’ (European Commission, 2024). This operational definition frames our inquiry into monitoring technologies initially developed for people with disabilities, whose dual-use implications remain ethically underexplored. Disability provides a crucial connecting thread in our analysis. On the one hand, technologies developed to support people with disabilities demonstrate how inclusive design fosters accessibility and care in civilian contexts. On the other hand, the very same design features reveal the risks of dual use, since assistive infrastructures may be reappropriated in ways that contribute to surveillance, restriction, or even coercion.

In this paper, we explore monitoring technologies initially aimed at people with disabilities, understanding that in extreme contexts, such as armed conflict or emergencies, any individual can acquire a temporary or permanent condition of disability or incapacitation. In these circumstances, the ability to request assistance in alternative or adaptive ways can make a decisive difference in the survival and well-being of these individuals. Therefore, there are reasons to consider that data generated in civilian environments, presumably intended to improve care and support for people with disabilities, can also serve as a basis for developing rapid response and coordination systems in defensive scenarios, and vice versa.

Despite strict regulations on the conception, design, development, and deployment of technologies in both the defense and civilian sectors [Trustworthiness for AI in Defence (TAID), 2025; European Union, 2024], the dual-use domain remains underexplored and insufficiently regulated. At this point, it is important to distinguish between AI as a general-purpose technology and AI as a product implemented by governments or institutions. General-purpose AI models, such as large language models or multimodal architectures, are designed for transversal use across multiple domains. The EU AI Act dedicates an entire title to their regulation, while simultaneously explicitly excluding defense from its scope. By contrast, when AI is embedded in specific governmental products, such as population surveillance or medical triage systems, it ceases to be merely general-purpose and becomes an applied instrument of governance.

The dual-use dilemma involves not only the risks of military appropriation of civilian systems but also the potential for defense-driven innovations to be redirected toward beneficial civilian purposes, such as in contexts of disability, emergency response, or public health. This distinction is essential for understanding how dual-use emerges not only from technical architectures but also from the political and regulatory contexts in which AI is deployed.^1^ Within this dual-use domain, it is essential to adopt a balanced perspective that recognizes both sides of the equation: defense-driven technologies can significantly improve civil emergency response, but without clear and enforceable oversight measures, those same technologies risk being used in ways that undermine public welfare.

This balanced approach does not seek to disregard or trivialize the associated ethical or political dilemmas, but instead emphasizes that establishing clear oversight parameters and ensuring their adherence throughout the technology lifecycle is the only viable way to guarantee benefits for the civilian population, especially if robust ethical safeguards are integrated from the outset. Furthermore, this perspective aims to contribute to the development of specific regulations for dual-use technologies while also establishing ethical measures to ensure that neither development nor innovation are hindered nor fundamental human rights are violated in the pursuit of progress.

In this article, we adopt thus an integrative perspective that examines these challenges and opportunities simultaneously. First, we address the conceptualization of the dual-use dilemma, exploring how technologies initially designed for civil monitoring can be adapted or repurposed for defensive applications, with a particular focus on the associated ethical risks. Next, from a procedural perspective, we analyze the critical importance of technical and ethical decisions made during the initial design of these technologies and how these decisions impact their subsequent use in very different contexts. We chose to mainly adopt a procedural perspective because our central interest lies in examining how early design decisions shape the ethical trajectories of AI systems, particularly when they are later reappropriated for dual-use purposes. Focusing on procedures allows us to highlight the role of design choices as elements that determine whether these systems can be inclusive and safe for vulnerable users. This perspective provides an intuitive starting point: since dual-use risks often originate in design features that persist unchanged across civilian and defense applications, analyzing these decisions from a procedural lens enables us to expose both the ethical potential and the vulnerabilities of aid systems. To conclude the paper, we highlight the need for proposals for the development of specific ethical strategies, focused on adapted prompting techniques, to minimize risks and maximize societal benefits, particularly in situations where individuals in vulnerable states must seek help in highly complex contexts.

Although our analysis focuses on the European regulatory framework, it is important to acknowledge that the development of AI models and infrastructures is intrinsically internationalized. This fact has become particularly evident in initiatives such as ReArm Europe and Europe Readiness 2023, where the urgency of securing European AI chip manufacturing exposes a deeper tension: while the EU seeks sovereignty through regulating AI internal frameworks, the very material basis of these technologies is entangled with global supply chains that serve both civil and defense purposes. In this sense, the dual-use concern is not only about the technical reappropriation of civilian systems for defense ends (aka dual-use dilemma, cf. infra. Sec. 2.2), but also about the geopolitical dependencies that arise when AI models or chips designed abroad are embedded in European infrastructures. These dependencies underscore how the civil–defense divide falls outside the scope of current European regulations, highlighting the need for governance strategies that can address dual use as both a technological and geopolitical condition.

The rest of the article is organized as follows. In section 2, we examine the conceptual foundations of the dual-use dilemma, highlighting how surveillance technologies developed to support people with disabilities can be used in defense contexts. Section 3 focuses on the procedural perspective, analyzing how initial design decisions shape the ethical trajectory of assistive systems and illustrating their dual-use potential through concrete alert modalities and technical applications. Section 4 broadens the debate by introducing the notion of implementation bias, emphasizing the risks and ethical challenges that arise when systems migrate from civilian to military environments. Finally, in Section 5, we conclude by outlining ethical incentive strategies aimed at minimizing risks and maximizing social benefits, especially in contexts where vulnerable individuals must seek assistance under extreme conditions.

## 2 Dual-use environment

Dual-use technologies are traditionally understood as those that can be applied to both civilian and defense purposes, as well as those that can be used for both legitimate and illegitimate purposes (Miller, 2018; NATO Parliamentary Assembly, 2024; Sans Pinillos and Vallverdú, 2025).^2^ Classic cases include nuclear energy and biotechnology (Miller, 2018; Selgelid, 2009). For instance, the industrial production of ammonium nitrate has clear civilian applications, yet also has the potential for destructive uses (Forge, 2013). These examples illustrate how dual-use is not an abstract category but a practical condition of many contemporary technologies. However, although dual-use currently refers to a particular way of conceiving and designing, its meaning is not far removed from the idea that technology (from the Greek τέχνη (*tékhnē*), used to mean ‘knowledge of how to make things’) is never neutral (Stiegler, 1998; Reijers et al., 2025, p. 9).

Aristotle (2009)^3^ already noted that any techne aims to bring into being something that does not yet exist, with its causal principle lying in the producer (from the Greek ποιεῖν (*poiesis*), which means ‘to make’) rather than in the product itself. Heidegger (2008) builds on this by arguing that, beyond mere instrumentality, technics “brings-forth” what was previously hidden: even if a technical object draws its motive force from elsewhere, it nonetheless effects a shift from concealment to disclosure, thereby constituting a distinct mode of truth. From this perspective, a dual-use technology always makes visible certain capacities (for example, adaptive communication or emergency response) while simultaneously keeping other capacities in reserve (such as lethal targeting or coercive control). In other words, its very design *reveals* an ambivalent field of possibilities, confirming that the dual nature of such technologies is not a mere regulatory category but arises from the essence of technology itself.

This theoretical perspective enables us to understand why technologies often reveal both legitimate and illegitimate capabilities, depending on their context of use. As illustrated by cases such as nuclear energy and biomedicine, the same underlying technē discloses dual-use potentials. Building on this lineage, we now focus on monitoring technologies applied to disability, which likewise reveal both care-oriented and coercive possibilities.

The first related case studies were nuclear energy (Miller, 2018) and biomedical research (Esposito, 2005), both of which have proven uses in civilian and military sectors. To date, several sectors have already been analyzed from this perspective of dual-use technology. In this work, we focus on monitoring technologies applied to individuals with disabilities, which can serve to coordinate rapid and accurate assistance and as a targeting process for uses that transcend the personal benefit of the persons being monitored.

### 2.1 The notion of disability

Let us emphasize that the concept of disability is neither static nor unequivocal; thus, some clarification is necessary when studying the case of this paper. Certainly, the term undoubtedly evokes deep and multifaceted questions regarding its ontological status, its ethical and moral implications, as well as its relevance to other domains like political philosophy and the social sciences (Vehmas and Riddle, 2019). The field of disability studies, that is, an interdisciplinary area drawing on philosophy, medicine, and the social sciences, provides organized theoretical frameworks for understanding disability (for a general overview, see Watson et al. (2019)); and even if these frameworks do not yield a single, definitive interpretation of the term, they support analytical engagement by delineating key elements of the discourse. In this way, disability has traditionally been framed through distinct but often complementary perspectives, two of which have traditionally been highlighted as seminal: the medical model and the social model. The medical model emphasizes bodily impairment and clinical intervention, treating disability primarily as a problem to be remedied through diagnosis, treatment, or rehabilitation (Fisher and Goodley, 2007; World Health Organization, World Bank, 2011); that is, this model focuses on diagnosis and physical remediation. In contrast, the social model shifts attention to structural and environmental barriers that restrict participation, arguing that disability is produced as much by social exclusion as by individual conditions (Oliver, 1990; Barnes and Mercer, 2010). In other words, this model situates disability within social, environmental, and policy barriers.

Integrating both models establishes a foundation for analyzing assistive technologies not merely as corrective tools but as mediators of social participation and autonomy. Indeed, a sociomedical perspective builds on this integration by acknowledging both the medical realities of impairment and the societal contexts that shape how impairments become disabling (Shakespeare, 2014). From a sociomedical standpoint, disability is not static: it may be permanent, progressive, or temporary, depending on medical circumstances and social support. This recognition is particularly evident in rehabilitation medicine, where assistive devices are often deployed during recovery phases [for example, after strokes or orthopedic injuries (Kairalla et al., 2016)]. Similarly, research on wartime veterans highlights how impairments caused by armed conflict can lead to lifelong disability (Karmarkar et al., 2009) but can also involve temporary reliance on assistive technologies during rehabilitation and reintegration (Lowe et al., 2024). Moreover, veterans transitioning from acute injury to civilian life often face unique housing and support challenges (Wilson et al., 2020).

Indeed, by situating technology within both clinical and social frameworks, it becomes more clear that assistive devices are not merely mechanical aids, but mediators of autonomy, inclusion, and identity. This perspective underscores the importance of considering assistive technologies across a continuum of disability experiences (from temporary to permanent) and in diverse social contexts, including those of veterans, aging populations, and people with chronic conditions. Recent systematic reviews illustrate the evolving scope of assistive technologies, from early mechanical aids to robotic and user-centered designs (Zallio and Ohashi, 2022).

However, this hybrid perspective on disability does not capture all its uses and meanings in practice. Accordingly, and in line with the perspective adopted in Costa (2025), this paper adopts a relatively inclusive and broader definition of disability. While it does not aim for an exhaustive conceptual analysis, it partially aligns with the foundational ideas of the social model of disability and incorporates medical aspects from the sociomedical model. On this basis, we include in our analysis research that examines individuals identified as persons with disabilities or those falling under the purview of the sociomedical model.

Regarding categories of disability, we follow World Health Organization, World Bank (2011), which does not adopt rigid classifications but identifies several interrelated categories that reflect the diverse ways in which health conditions can limit functioning and participation. These categories include impairments (problems in body function or structure), activity limitations (difficulties executing tasks or actions), and participation restrictions (problems engaging in life situations), all of which are shaped by interactions with environmental and personal factors. While the arguments presented in this paper are relevant across all categories of disability, those related to cognitive functions, sensory impairments (especially deafness and blindness), and mobility restrictions requiring auxiliary devices are particularly sensitive to the issues discussed.

Considering dual-use capacity is inevitable, both epistemological and moral, as we are currently in a scenario where all technology will eventually become ubiquitous and intrinsically dual-use. Certainly, this statement was uttered at the end of last year by Manuel Heitor, chair of the European Commission’s high-level group for Horizon Europe and the forthcoming Framework Program 10, when he said that it no longer makes sense to identify and classify technologies as dual-use or not (Greenacre and Zubașcu, 2024). The start of 2025 has only served to highlight this fact, with proposals such as the ReArm Europe plan (White Paper for European Defence and the ReArm Europe, 2025), an initiative aimed at achieving technological sovereignty in record time, whose one of its most notable strategies is the systematic integration of the civil sector into the European defense plan. In turn, this, aimed directly at reducing the technological gap with the rest of the global powers, implies a radical acceleration of the innovation cycle and a very real risk that aspects of design, development, and deployment will not guarantee functions whose use will not trigger unethical consequences (Taddeo, 2025, p. 16). The situation is further exacerbated by the dual-use nature of current and emerging technologies, as their development spans both civilian and military domains, ultimately aiming to create a techno-productive ecosystem adaptable to a wide range of applications, from social welfare to tactical deterrence. Furthermore, bearing in mind that there are functions that can generate different consequences depending on the context of use, it is important to distinguish between the use itself and the purpose for which it is applied. The concept of monitoring, for example, takes on dual-use nuances when analyzed in the civil and defense spheres due to its technical architecture and its ability to be adapted to different purposes. Technologies such as multi-object tracking, used in research and development for the simultaneous tracking of multiple people or objects, have proven useful in care settings, such as monitoring people with disabilities in urban or home environments.

However, the same type of technology could be applicable to security or defense surveillance contexts, whether with drones or other unmanned systems, especially when data collected in civilian settings and under peaceful conditions is later repurposed in wartime or during states of emergency. In such scenarios, classifications based on disability or other forms of vulnerability may shift from serving care-oriented goals to enabling selective targeting, discrimination in the distribution of critical resources, or the reinforcement of social hierarchies. Across political systems, the aggregation and instrumentalization of personal data have contributed to practices of marginalization, forced segregation, and, in extreme cases, forms of systemic violence. In relation to the latter, it has been shown that, historically, people with disabilities have been particularly affected (Figueroa et al., 2023).

These risks become particularly salient when such dual-use capabilities are embedded in systems driven by AI prompting architectures, especially those originally developed to support vulnerable populations, such as persons with disabilities. Technologies that automate care-related decision-making—through voice-based commands, contextual prompting, or adaptive assistance—may also lay the groundwork for military command structures or operational targeting systems. In this regard, let us recall the four fundamental steps of the OODA loop (Observe, Orient, Decide, Act), which encapsulate the decision-making process of Lethal Autonomous Weapon Systems (LAWS) and will be used in the arguments presented in this paper. Initially introduced by Boyd (2018) in the context of general military strategy, these steps are defined as follows: Observe (collect available data and detect potential targets), Orient (recognize and identify the targets), Decide (evaluate and determine whether to engage the targets), and Act (execute engagement with the targets). The shift from assistive prompting to strategic automation reveals how design choices made in peaceful, civilian contexts can carry over into high-risk domains, often without the ethical safeguards that such transitions would require. This shift in use context is what lies at the heart of the dual-use dilemma explained in the next section.

Assuming that prompting is itself a technology, it becomes urgent to clarify how our proposal can be reconciled with institutional logics and aligned with the cross-cutting nature of dual use. This issue directly intersects with the interests and priorities of diverse agendas and business models, which inevitably shape people’s lives. Thus, although it may sound like a tautology, it is essential that ethical prompting be understood as an ethical matter of institutional coordination and governance.

### 2.2 Dual-use dilemma in AI

The dual-use context becomes particularly sensitive when beneficial uses—and, we would add, legal and legitimate uses in the context of war^4^—have the potential to cause harm (World Health Organization, 2020). We refer to the *dual-use dilemma*, namely, a situation in which an agent has moral reasons to perform two actions (or more), but cannot perform both, thus being condemned to moral failure (whatever they do, they will do something wrong, cf. McConnell, 2022, p. 2), which arises when technology or knowledge conceived for legitimate and beneficial purposes becomes a tool for causing harm (Miller and Selgelid, 2007). Then, partial automation of the OODA cycle’s *Observe*/*Orient* phases via civilian infrastructures compresses timelines and lowers redeployment costs.

Unlike “classic” dual-use areas mentioned above, AI acts less as a discrete technology than as an integrating force across all technology stacks. As we stated at the introduction, most emerging technologies are increasingly seen as dual-use by nature, but AI intensifies this condition: reprogrammable models, monitoring tools, and simulation engines can be reused almost instantly for risk detection, where detected risks can be programmed at any time. This dual use has become even more evident as warfare in urban environments becomes increasingly common, where civilian infrastructure and populations constitute the main terrain of observation (King, 2025). In this sense, the role of AI in dual use is not incidental but constitutive, as its ability to permeate and reconfigure other technologies directly lowers the threshold for its reuse in civilian and defense contexts. Beyond the “pure” technical layer, data-integration platforms (e.g., Palantir-type systems) operate across public health, critical infrastructure and security/defense decision-support, normalizing near-seamless migration between civilian and military uses under the guise of being protectors concerned with preserving shared values (Vlassis, 2024; Tan, 2025).

The dual-use context is best understood as emerging from a network of interactions between technology and its stakeholders, encompassing both propositional knowledge (“know-what”) and practical skills (“know-how”). Rather than treating technology as a static artifact, this view situates it within a dynamic lifecycle (from innovation and design through development, deployment, testing, distribution, and use) where every phase shapes its possible applications (cf. Tucker, 2012, p. 1).

This perspective coincides with established descriptions of technology as inherently value-laden and multifunctional (de Vries, 2005; Reijers et al., 2025), illustrating that any system can produce both beneficial and harmful outcomes, depending on how and by whom it is used. High-resolution camera drones are a clear example: while they can contribute to precision agriculture by monitoring crop conditions, the same sensors and algorithms can be repurposed for military surveillance or target tracking (Grossman, 2013). Therefore, decisions made during the early stages of design (about sensor capabilities, data governance, and user interfaces) have an ethical weight in defining which uses will most easily materialize during deployment (Sans Pinillos and Vallverdú, 2025).

Recent and ongoing conflicts demonstrate how these dynamics materialize in practice. In Gaza, drones have long been used not only as weapons but also as instruments of persistent surveillance (Rogers, 2014), producing an environment of continuous real-time observation over civilian populations that endures to this day. Likewise, in Ukraine, low-cost FPV drones originating in the civilian market were rapidly adapted for reconnaissance, artillery correction and strike roles, and even off-the-shelf civilian drones have been employed (Czerwiński and Balcerzak, 2024). Both examples highlight how the OODA cycle’s observation and orientation phases can be accelerated through civilian infrastructures, thereby reinforcing the dual-use dilemma.

To conclude this section, we emphasize the importance of ethical considerations throughout the development of dual-use technologies. By framing dual use in terms of stakeholder interactions and lifecycle decisions, we uncover the practical and moral dilemmas that arise when civilian technologies migrate to defense applications without adequate oversight. This approach highlights that ethical responsibility does not begin with implementation alone but is embedded throughout the evolution of technology, requiring continuous reflection on who controls data, under what legal frameworks, and with what accountability mechanisms to prevent harmful reuses.

From the perspective of the OODA cycle, the relationship between institutional decisions and their dependence on sociocultural ethical frameworks becomes particularly visible in the phases of “Observation” and “Orientation.” These stages not only involve technical processes of data acquisition and interpretation but also reflect normative boundaries that define which actions are regarded as legitimate and which are not. In China, for instance, instruments such as the Cybersecurity Law (Qi et al., 2018), the Data Security Law (Chen and Sun, 2021), and the military–civil fusion (Woods, 2025) underscore how security is emphasized over privacy. By contrast, in Europe, privacy and data minimization are legal imperatives under frameworks such as the GDPR and the EU AI Act, even in the TAID. The resulting asymmetry illustrates how dual-use technologies are embedded in divergent normative environments, where what is seen as an “ethical safeguard” in one context may be irrelevant, or even unintelligible, in another.^5^

## 3 The role of the assistant: ways of requesting help and adaptive response

In this paper, we define aid systems (whether a hardware device, software interface, or data-driven protocol) through which individuals with disabilities or those experiencing temporary vulnerability can generate alerts to request assistance and receive a timely response.

### 3.1 Alert modalities and their dual-use potential

Aid alerts can take multiple forms depending on user needs and operational constraints. Below is a list showing how each modality is used in civilian assistance and how it translates unmodified into a military or defense setting (National Academies of Sciences, Engineering, and Medicine, 2018). In all cases, these alert modalities rely on the same basic components (microphones, vibration motors, GPS modules, and secure messaging) regardless of whether the end user is a civilian at home or a soldier on the battlefield.

#### Auditory alerts

- Civilian: A distinct tone or synthesized voice message (e.g., “Help needed”) notifies nearby caregivers, family members, or emergency services.
- Military: The same audio cue can alert combat medics or a squad leader that a soldier is wounded and needs extraction.

#### Tactile/haptic alerts

- Civilian: Vibration patterns on a wearable wristband confirm that an alert has been received, especially useful for hearing-impaired or deaf users.
- Military: Identical vibration codes on a soldier’s wearable (vest or belt) can convey discrete instructions such as “Proceed to rally point” or “Medic needed” when radio silence is required.

#### Text messages/visual notifications

- Civilian: Automated SMS or push notifications (via cellular or satellite) transmit geolocation, user ID, and a brief description (e.g., “User fell at coordinates X, Y; heart rate elevated”) to family or a 112/911 dispatcher.
- Military: The same message, with identical fields, is rerouted to a Forward Operating Base’s tactical operations center, triggering a MEDEVAC or re-routing allied forces to secure that grid coordinate.

#### Notifications to support staff or command centers

- Civilian: In a hospital ward or care facility, alert data feeds directly into the nursing station’s dashboard, activating internal response protocols (e.g., dispatch a floor nurse).
- Military: In a conflict or disaster zone, identical data streams feed a military command post. The command post uses that data to dispatch field medics, unmanned CASEVAC drones, or security forces without altering the underlying hardware or software stack.

### 3.2 Technical implementation and dual-use considerations

Effective aid solutions combine three essential components, each with dual-use potential in both civilian and military environments: critical situation detection algorithms, scalable alert escalation protocols, and adaptive user interfaces (Olson and Redkar, 2018).

#### 3.2.1 Algorithms for detecting critical situations

- Lightweight machine learning models (e.g., Liu et al., 2024) identify events like falls, distress signals, or non-habitual body postures.
- Inputs from accelerometers, gyroscopes, microphones, and RGB-D cameras or biometric sensors enable real-time analysis for health monitoring and for assessing personnel status in tactical scenarios.
- Anomaly thresholds, for example, acceleration exceeding 1.5 g within 0.2 s, trigger automatic alerts that apply both in home emergencies and in the field to detect a soldier down.

#### 3.2.2 Scalable alert escalation protocols

- Level 1 (Self-Verification): A brief haptic or audio prompt requests confirmation. This minimizes false alarms and allows a user in military scenarios to confirm without revealing their location, for example, via bone-conduction prompts.
- Level 2 (Notification to Nearby Network): If there is no response within 10–15 s, a preconfigured message is sent via SMS or encrypted network to local contacts such as family and caregivers in civilian use or combat medics and unit leaders in military use. The data package includes user ID, basic status information (e.g., whether the user is unresponsive or the battery status of the alert device), and GPS coordinates.
- Level 3 (Activation of Emergency or Operational Services): After an additional time limit, for example, 2 mins, the system automatically connects to emergency services, such as 911, or to a tactical operations center. This link transmits critical data such as minimal biometrics, medical history, and precise location, facilitating civilian emergency response or military MEDEVAC operations.

#### 3.2.3 Adaptive user interfaces

- Hearing-Impaired and Deaf Users: Use of vibration patterns, such as long versus short pulses and high-contrast visual cues. These features are also valuable in noisy combat environments.
- Visually-Impaired Users: Reliance on haptic feedback and synthesized voice prompts. In military scenarios, bone-conduction speakers deliver notifications without betraying the user’s position.
- Users with Cognitive Disabilities: Simplified interfaces with fixed pictograms, for example “Aid” and “I’m OK,” and concise messages to support rapid comprehension under stress.
- Users with Reduced Mobility: Voice commands such as “Aid” or “Call Contact” and easy-access physical buttons. In military operations, the same buttons can be reconfigured to send signals such as “evacuate” or “stand down.”

Combining precise detection, structured alert escalation, and adaptable interfaces, these aid systems create a reliable, robust communication infrastructure that minimizes both false positives and false negatives in detection, while supporting both home or institutional assistance and tactical coordination in defense settings.

## 4 Ethical dimensions of deployment bias in AI: from intended design to unintended use

For the purpose of this paper, we analyze debates around ethical concerns arising from the use, development, and deployment of AI systems through two complementary approaches frequently highlighted in the literature. We do not claim that these approaches exhaust the breadth of AI ethics as a whole, but rather that they provide a pragmatic framework for examining the concrete ethical challenges that emerge in relation to existing AI systems. In particular, we follow the two-approach framework proposed by Ferrer Aran et al. (2021): the relational approach, which emphasizes biases present in datasets or algorithmic outcomes, and the procedural approach, which focuses on the design choices and decision-making processes involved in building AI systems (that is, the *logic* of the model). Each approach helps identify different types of bias (Suresh and Guttag, 2021; Balayn et al., 2021), such as *historical bias* in the case of the relational approach, or *aggregation bias* in the case of the procedural approach. Let us clarify that the notion of bias is understood as systematic distortion (among others, here we are referring especially to cognitive and computational ones) that affects the fairness, inclusivity, or intended function of an AI system, whether they originate from design decisions (procedural approach) or from the socio-political conditions under which systems are deployed (relational approach).

Beyond concerns about unintended consequences or biases from the procedural approach, the dual-use dilemma also includes cases of deliberate reappropriation, which would be related to the *deployment bias* (Suresh and Guttag, 2021; Balayn et al., 2021). This kind of bias stems from a discrepancy between the problem an AI system is originally designed to address and how it is ultimately applied by some users or within some contexts. For example, in these scenarios, civil data infrastructures originally designed for the care and assistance of people with disabilities might be strategically integrated into defense systems under changing geopolitical conditions.^6^ Thus, the problem is not that these systems malfunction or discriminate by both cognitive and curator bias [as was, for example, the case with the Correctional Offender Management Profiling for Alternative Sanctions, COMPAS, (Dressel and Farid, 2018)], but that they operate exactly as designed, albeit for different or even radically different purposes. Another example is about biometric gathered data intended to facilitate assistance in times of peace, which can have a main role in the OODA loop steps– especially in the first three–and may become, in a conflict context, a means of classifying, tracking, or reducing access to resources, or even, in the most extreme cases, a tool for targeting and eliminating individuals based on their supposed social utility or the probability of short-or medium-term survival.

This form of functional appropriation underscores the ethical urgency of critically examining not only how technologies are designed, but also who maintains control over the data, under what legal frameworks, and with a view to what future contingencies will determine the uses of their deployment. As mentioned before, these types of risks can be related to what is known in the technical literature as *deployment bias*. Unlike more traditional biases, which are usually found in algorithm design or dataset construction, deployment bias arises when a system designed, trained and validated under certain conditions is ultimately used in a different environment, where its effects are not only unforeseen but may be systematically harmful to social groups. This discrepancy becomes more pronounced in contexts of conflict or exception, such as states of war or emergency, where systemic functionalities are deployed without critical adaptation, reinforcing dynamics of control and coercion. Technologies originally designed for assistance or care, such as those used to monitor people with disabilities, can thus be repurposed for exclusion, movement restriction, or even selective identification and elimination based on criteria of military or political utility. In short, rather than representing a malfunction, this shift reflects a functional continuity with a reoriented purpose. In this case, let us insist and recall that the bias does not originate from a technical failure, but rather a functional dissonance between the environment for which the system was calibrated and the one in which it ends up being applied (Suresh and Guttag, 2021; Balayn et al., 2021).

In Section 3, a series of possible configurations for aid systems has been presented through a dual-use approach and illustrated within the context of a conflict scenario. Although potential uses of these systems have been considered, the primary focus has been placed on the design elements of the models; thus, the analysis has adopted a procedural approach. This perspective appears to be one of the most intuitive initial ways to examine the ethical concerns and challenges posed by AI systems, as also reflected in the literature. For instance, in relation to the environment considered in the present work, lethal autonomous weapon systems (LAWS) have been analyzed (Costa, 2025) from a procedural standpoint, incorporating a disability perspective. Indeed, certain factors to consider in the design of LAWS are also relevant to the analysis of aid systems, such as the inability of some users with disabilities to follow instructions needed to interact effectively with the system, or concerns related to the use of biometric data.

In this paper, we argue that adopting a dual-use perspective guides the analysis in two key directions. On the one hand, this dual-use environment compels us to recognize both the urgency and the positive necessity of shifting the debate from a purely procedural perspective to a relational one. Indeed, the socio-political context in which aid systems are deployed may distort their original design objectives, potentially resulting in unintended uses that reinforce discrimination against people with disabilities. As mentioned at the beginning of this section, one such example is the restriction of access to resources for these social groups. This scenario exemplifies not only a relational issue but also a categorical case of deployment bias.

Furthermore, the lack of control over data collected by aid systems in conflict scenarios and the subsequent construction of datasets raise additional concerns from a relational standpoint. These concerns encompass not only the use of the systems but also the design and composition of the datasets on which they are based. This overview shows that it is not only necessary to consider how monitoring technologies are designed, but also who controls their data, under what circumstances it is accessed, and what future uses, legal or otherwise, may be activated depending on the socio-political context in which they are deployed.

However, this perspective also supports a positive interpretation in the analysis of aid systems. First, the deployment, use, and experience of aid systems in conflict scenarios may provide valuable opportunities to significantly improve their design for everyday civilian contexts. For instance, insights gained from these settings could inform enhancements to specific design elements that are better suited to users with disabilities. Second, the data collected in such contexts could eventually be used to construct datasets that support analyses and studies aimed at better understanding the specific needs of people with disabilities when interacting with AI systems.

On the other hand, the dual-user perspective requires us to take into account scenarios that are more critical, complex, and potentially hazardous, such as those encountered in war settings. This highlights the need to nuance the concept of reappropriation discussed in this paper, as it can occur across different scales of severity. For instance, although the presence of biometric data poses significant challenges in both civilian environments and military or conflict zones, it can generally be argued that the latter face more sensitive and acute risks.

In conclusion, addressing deployment bias in AI requires an integrated ethical approach that bridges both procedural and relational perspectives, particularly in dual-use scenarios. By recognizing how socio-political dynamics shape the reappropriation of aid systems, we can better anticipate and mitigate the risks of harm to vulnerable populations like people with disabilities, while also identifying pathways for responsible and inclusive technological development.

## 5 Conclusions: toward ethical prompting strategies in conflict scenarios

In this paper, we have highlighted the dual-use dilemma inherent in monitoring technologies initially developed for individuals with disabilities, particularly emphasizing the risks of deployment bias in contexts of armed conflict or emergency situations. This exploration underscores that the complexity inherent in designing multimodal systems must ultimately serve effectiveness—specifically, enabling swift and reliable requests for assistance and ensuring appropriate responses in critical moments.

Furthermore, we argue that fostering ethically robust technological infrastructures requires integrating adaptive prompting techniques within the broader governance framework of dual-use technologies. This approach promotes systems capable of swiftly delivering effective assistance without inadvertently facilitating discriminatory or harmful practices. Ultimately, ethically-informed prompting becomes an essential part of responsible innovation, ensuring technology remains supportive rather than coercive, protective rather than exclusionary, across all scenarios.

Prompting is the practice of crafting input, commonly referred to as a prompt, that guides the behavior of a model (Liu et al., 2021). It involves instructing the model in natural language (or sometimes in structured formats) to perform a specific task or generate content. In this section, we illustrate, through the lens of dual-use technologies, how the form of prompts can help mitigate deployment biases in AI systems toward people with disabilities.

Prompting techniques vary in complexity and structure, offering different ways to guide models toward desired outputs (Wei et al., 2023; Zhou et al., 2023; Liu et al., 2021). Let us first recall different kinds of prompting. In this way, zero-shot prompting involves giving the model a task without any prior examples, relying solely on the phrasing of the prompt. For example, asking a model to “Translate ‘Good morning’ to Catalan.” In one-shot prompting, a single example is included to demonstrate the expected format, like “Translate: Hello → Bon dia. Now translate: Good morning →.” Few-shot prompting extends this by providing several examples to better shape the model’s behavior, as seen in “Translate: Hello → Bon dia. Goodbye → Adéu. Please translate: Thank you →.” For more complex reasoning, chain-of-thought prompting encourages the model to explain its reasoning step-by-step. For example, “Let us reason step by step.” Structured prompting uses formats such as templates or bullet points to constrain output, while instruction prompting clearly states what the model should do, like “Summarize the following paragraph in one sentence.” Finally, least-to-most prompting breaks down a complex task into subtasks, guiding the model through sequential steps to improve performance and interpretability.

Certainly, the rapidity and reliability of the aid systems we have exemplified in this paper derive primarily from optimized and ethically informed design rather than the mere addition of complex multimodal features. Consequently, the objective is to streamline alert modalities and escalation protocols to function robustly across varied scenarios while reducing ambiguity or potential misuse. Therefore, ethical prompting emerges as a fundamental strategy, helping clearly delineate how data and prompts should be structured to reduce risks associated with deployment bias, particularly toward vulnerable populations like individuals with disabilities.

In this work, we claim that some of these techniques are more appropriate to make explicit potential risks related to deployment bias, and focus the analysis on discrimination against individuals with disabilities.

The arguments presented in this paper suggest the necessity of developing specific single-type prompts. Such prompts, characterized by their simplicity, facilitate rapid and secure assistance responses, particularly valuable for incapacitated users within strictly defined defense operational domains. This approach explicitly acknowledges dual-use potential, highlighting direct applicability and substantial benefits for civilian populations with varying degrees of disability, thereby enhancing accessibility and reliability in both military and civilian emergency contexts.

## Data Availability

The original contributions presented in the study are included in the article/supplementary material, further inquiries can be directed to the corresponding authors.

## Footnotes

^1^As declared in the White Paper on Options for Enhancing Support for Research and Development Involving Technologies with Dual-Use Potential (European Commission, 2024), the European Commission has repeatedly acknowledged these regulatory shortcomings. The 2021 Action Plan on Synergies between Civil, Defence and Space Industries already identified the need for a gap analysis to improve complementarity between EU programs from R&D to deployment (European Commission, 2021; European Commission, 2022a). The 2022 Roadmap on Critical Technologies for Security and Defence further recognized that no framework currently exists for direct support of dual-use activities (European Commission, 2022b), while the Joint Communication “Defence Investment Gaps Analysis and Way Forward” explicitly called for amendments to strengthen synergies between civil and defence instruments (European Commission, 2022c).

^2^Apart from the obvious distinction between the civilian and defense sectors, the literature often frames dual-use in terms of beneficial/good vs. harmful/bad purposes (c.f., Selgelid, 2009). However, building on our previous work (Sans Pinillos et al., 2025), in defense contexts the contrast is more complex because not all “non-beneficial” uses are illegitimate, since preventing or neutralizing threats may not yield direct benefits. Selgelid already notes that research with dual-use potential involves “legitimate uses (e.g., medicine)” that might be diverted by malevolent actors for nefarious purposes (such as bioterrorism in the life sciences). Here we refine this point by differentiating between legitimate uses (which may be beneficial or merely non-beneficial) and illegitimate misuses (typically bad and/or harmful, as Selgelid introduces in his work).

^3^“All art [*tékhnē*] is concerned with coming into being, i.e., with contriving and considering how something may come into being which is capable of either being or not being, and whose origin is in the maker and not in the thing made; for art is concerned neither with things that are, or come into being, by necessity, nor with things that do so in accordance with nature (since these have their origin in themselves). Making and acting being different, art must be a matter of making, not of acting. And in a sense chance and art are concerned with the same objects; as Agathon says, ‘Art loves chance and chance loves art’” (EN Bk. 6, 4. 1140a11-20).

^4^This nuance is important because much of the current discourse on misuse tends to equate it solely with military or defense applications, overlooking the fact that such uses are often governed by jurisprudence, operational rules, and sociopolitical imperatives that demand thorough investigation and accountability. Assuming that both civilian and military sectors are susceptible to misuse, this paper highlights some of the associated risks. However, it also emphasizes that certain risks (especially those that occur within the boundaries of legality or even moral legitimacy in defense contexts) may be more urgent to examine if we are to formulate appropriate ethical and policy recommendations for the deployment of today’s disruptive and inherently dual-use technologies.

^5^This divergence does not call for moral adjudication so much as it highlights a broader philosophical question: to what extent is our own approach to “ethical prompting” inherently shaped by European values? What may appear as a universal framework for ethical safeguards could, in practice, be contingent on cultural, institutional, and legal traditions, raising the possibility that such an approach is not globally translatable but rather specific to Europe’s normative ecosystem.

^6^E.g., During the COVID-19 pandemic, various governments and private actors repurposed technological infrastructure (such as mobile apps, wearable devices, and geolocation technologies) originally designed for civil services, quickly integrating them into population surveillance systems for health and mobility control purposes, raising ethical concerns about privacy and civil rights (Donelle et al., 2023).
